# Maternal high fat diet during pregnancy and lactation alters hepatic expression of insulin like growth factor-2 and key microRNAs in the adult offspring

**DOI:** 10.1186/1471-2164-10-478

**Published:** 2009-10-16

**Authors:** Junlong Zhang, Fang Zhang, Xavier Didelot, Kimberley D Bruce, Felino R Cagampang, Manu Vatish, Mark Hanson, Hendrik Lehnert, Antonio Ceriello, Christopher D Byrne

**Affiliations:** 1 Clinical Science Research Institute, Warwick Medical School, Clinical Sciences Building, University Hospital - Walsgrave Campus, Clifford Bridge Road, Coventry, CV2 2DX, UK; 2 Department of Statistics, University of Warwick, Coventry, CV4 7AL, UK; 3 Institute of Developmental Sciences, Developmental Origins of Health and Disease Division, University of Southampton Medical School, Southampton General Hospital, Southampton, SO16 0YD, UK; 4 Albert Einstein College of Medicine, 1301 Morris Park Avenue, Bronx, New York, NY 10461, USA; 5 1st Medical Department, University of Lübeck Medical School Ratzeburger Allee 160, 23538 Luebeck, Germany; 6 Chair of Endocrinology, University of Udine, Italy

## Abstract

**Background:**

miRNAs play important roles in the regulation of gene functions. Maternal dietary modifications during pregnancy and gestation have long-term effects on the offspring, but it is not known whether a maternal high fat (HF) diet during pregnancy and lactation alters expression of key miRNAs in the offspring.

**Results:**

We studied the effects of maternal HF diet on the adult offspring by feeding mice with either a HF or a chow diet prior to conception, during pregnancy and lactation, and all offspring were weaned onto the same chow diet until adulthood. Maternal HF fed offspring had markedly increased hepatic mRNA levels of peroxisome proliferator activated receptor-alpha (ppar-alpha) and carnitine palmitoyl transferase-1a (cpt-1a) as well as insulin like growth factor-2 (*Igf2*). A HF diet induced up-regulation of ppar-alpha and cpt-1a expression in the wild type but not in *Igf2 *knock out mice. Furthermore, hepatic expression of let-7c was also reduced in maternal HF fed offspring. Among 579 miRNAs measured with microarray, ~23 miRNA levels were reduced by ~1.5-4.9-fold. Reduced expression of miR-709 (a highly expressed miRNA), miR-122, miR-192, miR-194, miR-26a, let-7a, let7b and let-7c, miR-494 and miR-483* (reduced by ~4.9 fold) was validated by qPCR. We found that methyl-CpG binding protein 2 was the common predicted target for miR-709, miR-let7s, miR-122, miR-194 and miR-26a using our own purpose-built computer program.

**Conclusion:**

Maternal HF feeding during pregnancy and lactation induced co-ordinated and long-lasting changes in expression of *Igf2*, fat metabolic genes and several important miRNAs in the offspring.

## Background

miRNAs are small (~21 nt) non-coding RNAs that were originally discovered to regulate development in *C. elegans *[1-3]. A significant number of miRNAs are conserved across different species [4-7]. miRNAs regulate gene functions mainly through degradation of their cognate mRNAs by perfect matches with the mRNA molecules; or via inhibition of protein translation through base pairing of ~7 nucleotides (called "seed sequence") between miRNA and the 3'-untranslated region (3'-UTR) of the target mRNA molecules [8]. Expression of miRNAs may be regulated by transcription factors (e.g. myogenin and myoD regulate expression of a number of miRNAs [9]), and transcription factors *per se *may also be regulated by miRNAs (e.g. miR-1 promotes myogenesis by targeting histone deacetylase 4, a transcriptional repressor of muscle gene expression) [10]. A single miRNA can repress the production of hundreds of proteins, but this repression is relatively mild [11]. On the other hand one mRNA can be targeted by several miRNAs, which have additive effects in regulation of protein synthesis [12]. For example, SMAD-1 gene has two predicted binding sites for miR-26a [12], and greater suppression effects on protein translation have been observed in mRNAs containing multiple binding sites for a miRNA [13].

miRNAs are involved in the regulation of almost all important biological processes including development [14], differentiation, cell proliferation, cell cycle regulation [15,16] and energy metabolism [17], including fat metabolism and glucose homeostasis [18,19]. For example, miR-375 suppresses glucose-induced insulin secretion in pancreatic β-cells [20], thus demonstrating an essential role in plasma glucose homeostasis. Knocking down of endogenous miR-122, a miRNA abundantly expressed in the liver, reduces plasma cholesterol concentrations in mice [21], with parallel up-regulation of 363 mRNA transcripts and down-regulation of 305 mRNA transcripts in the liver [21]. MiR-143 stimulates human adipocyte differentiation [22]. Analysis of global profiles of miRNA expression in skeletal muscle with microarray shows that expression of 4 miRNAs (miR-29a, miR-29b, miR-29c and miR-150) are up-regulated [23], whereas expression of 11 miRNAs (miR-379, miR-127, miR299-5p, miR-434-3p, miR-335, miR130a, miR-19b, miR-451, miR-148a, miR-199a and miR-152) are down-regulated in skeletal muscle of type 2 diabetic rats [23].

The prevalence of obesity is increasing markedly in industrialised countries [24-28], and high fat, high protein, low carbohydrate diets including proprietary diets such as the Atkins diet are widely consumed [29-31]. The prevalence of obesity in women of reproductive age continues to rise [32], and it is likely that many women of reproductive age also consume a low carbohydrate, high fat and high protein diet during pregnancy and lactation. However the effects of increased maternal dietary consumption of fat during pregnancy and weaning on the long term health of the offspring are not fully characterized.

Many studies have indicated long-term consequences of maternal dietary modifications (e.g. caloric or protein restrictions) during pregnancy and lactation on the development of insulin resistance and risk of cardiovascular disease in the offspring [33-37]. We have previously shown in mice that adult offspring of dams fed a low carbohydrate, high fat and high protein diet during pregnancy and lactation but weaned onto a chow diet have reduced hepatic triglyceride levels in association with increased protein levels of key genes regulating fatty acid oxidation including carnitine palmitoyltransferase-1a (CPT-1a) and peroxisome proliferator-activated receptor-alpha (PPARα) predominantly in the female offspring [33]. Pups born to dams on a high fat (HF) diet during gestation and lactation have increased percentage of body fat, plasma glucose, free fatty acids, insulin and cholesterol levels, liver weight and lipid concentrations at weaning or in adulthood [38,39].

Fetal growth is regulated by insulin-like growth factor 2 (IGF2) [40]. Recent data suggest that IGF2 may regulate fat metabolism. For example, body weight is affected by several polymorphisms in the *Igf2 *gene [41,42], and low circulating IGF2 concentrations are associated with weight gain and obesity [43]. In contrast, high circulating IGF2 levels associated with the Apal polymorphism of *Igf2 *are associated with low body weight in middle aged men [44]. Mice overexpressing *Igf2 *have increased fatty acid oxidation [45]. Maternal dietary protein restriction reduces hepatic expression of *Igf2 *in the male fetal offspring. However, whether maternal HF feeding alters offspring *Igf2 *expression has not been documented.

Following our previous studies on maternal high fat, high protein and low carbohydrate diet[33], we used a modified diet to investigate whether maternal HF feeding during pregnancy and lactation altered mRNA levels of *ppar-α *and *cpt-1a *and whether changes in *ppar-α *and *cpt-1a *were related to changes in *Igf2 *expression. We also analyzed global miRNA expression profile in the liver to determine which miRNAs were altered in the offspring born to dams fed a HF diet during pregnancy and lactation.

## Results

### Maternal HF fed offspring had increased mRNA levels of *ppar-α*, *cpt-1a *and *Igf2 *in the liver

We have previously shown that maternal high fat, high protein and low carbohydrate diet fed offspring had increased protein levels of PPARα and CPT-1a levels in the liver, in association with reduced hepatic lipid levels, despite having no significant changes in body weight, plasma glucose and lipid profile [33]. In this study, a modified HF diet was fed to dams, in which the percentage of fat was increased by more than 2-fold with a small increase in protein levels compared to the chow diet (see methods section). Consistently, no significant difference in body weight, fasting plasma triglyceride, total cholesterol and glucose levels were observed between maternal HF fed offspring weaned on a chow diet (HF/C) and control mice (C/C, data not shown). mRNA levels of *ppar-α *and *cpt-1a *in the HF/C mice were increased by ~1.6 and ~3.7-fold respectively compared to C/C mice (p < 0.05 and 0.01 for *ppar-α *and *cpt-1a *respectively, Table 1).

**Table 1:** Effects of maternal HF feeding on hepatic mRNA levels in adult offspring

**Genes**	**C/C (n = 7)**	**HF/C (n = 7)**
ppar-α	61873 ± 7638	97445 ± 11712*
cpt-1a	34193 ± 4420	126777 ± 23720**
*Igf2*	520 ± 70	1404 ± 266**

Levels of mRNA expression (arbitrary units) were measured with real time qPCR as described in the method section. Mean ± S.E. * p < 0.05 and ** p < 0.01.

As the maternal HF diet was implemented prior to conception and continued throughout pregnancy and lactation, we investigated whether expression of *Igf2*, an imprinted gene encoding a growth factor expressed during early development [40] was altered in the maternal HF fed offspring. The mRNA level of *Igf2 *was increased by ~2.7-fold in maternal HF diet fed offspring compared to the control animals (p < 0.01, Table 1).

To determine whether increased expression of *ppar-α *and *cpt-1a *was related to increased expression of *Igf2 *in the maternal HF fed offspring, we measured mRNA levels of *ppar-α *and *cpt-1a *in *Igf2 *KO mice. A HF diet modestly increased hepatic expression of *ppar-α *and *cpt-1a *in the WT mice (p < 0.05 and 0.01 for *ppar-α *and cpt-1a respectively, Table 2), but the HF diet had no effects on *ppar-α *and *cpt-1a *expression in the KO mice (p = 0.98 and 1.0 for *ppar-α *and *cpt-1a *respectively, Table 2), suggesting that expression of *Igf2 *was required for the HF diet induced up-regulation of expression of *ppar-α *and *cpt-1a*.

**Table 2:** Hepatic gene expression in wild type and Igf2 knock out mice

**Genes**	**KO-C (n = 5)**	**WT-C (n = 6)**	**KO-HF (n = 6)**	**WT-HF (n = 6)**
ppar-α	210.34 ± 33.17	220.32 ± 40.99	283.88 ± 15.97	370.91 ± 31.35*
cpt-1a	266.71 ± 17.41	236.68 ± 50.41	266.05 ± 36.59	391.26 ± 21.61**

Both the wild type (WT) and *Igf2 *knock out (KO) mice were fed either a HF or chow diet as described in the methods section. mRNA levels were measured using real time PCR. Mean ± S.E. * p < 0.05 and ** p < 0.01 (HF v chow diet).

### Hepatic expression of let-7c was reduced in maternal HF offspring

Let-7 was originally discovered due to its regulation of developmental timing in *C. elegans*, through binding to the 3'-UTR region of Lin-41 [46,47]. Levels of let-7c and other members of let-7 including let-7a, let-7b and let-7d were reduced by 2-2.5-fold in maternal HF fed offspring compared to the control animals (p < 0.01 for let-7a, let-7b and let-7d, Table 3).

**Table 3:** Hepatic let-7s levels in maternal HF fed offspring.

**Genes**	**Expression levels (arbitrary units)**	
	**C/C (n = 7)**	**HF/C (n = 7)**
**let-7c**	118.10 ± 9.71	60.31 ± 6.80**
**let-7a**	278.62 ± 15.64	107.64 ± 11.82*
**let-7b**	107.10 ± 5.45	45.82 ± 4.28**
**let-7d**	164.68 ± 15.50	80.29 ± 8.10**

Levels (arbitrary units) of let-7s were measured as described in the methods section. Mean ± S.E. ** p < 0.01.

Having observed reduced expression of let-7s in maternal HF fed offspring, we measured the global miRNA expression profile with microarrays.

### Expression of ~5.7% of miRNAs was altered in the maternal HF fed offspring

A cut-off threshold of 1.5-fold change [48] in miRNAs was used to determine whether altered miRNAs levels were likely to be significant. Of 579 miRNAs measured, expression of 10 miRNAs (~1.7%) was increased by ~1.5-2-fold (average increase was ~1.64-fold, Table 4), whereas expression of 23 miRNAs (~3.97%) were reduced by 1.51 - 4.93 fold (average reduction of 2.16-fold, Table 5), with miR-483* showing the biggest reduction (by ~4.9-fold, Table 5). In contrast, expression of most miRNAs remained unchanged (Additional file 1: Table S1).

**Table 4:** Hepatic miRNA levels increased in maternal HF fed offspring

**miRNAs**	**C/C**	**HF/C**	**↑ Fold**
miR-503*	12765	19148	1.50
miR-379	13463	20313	1.51
miR-770-3p	17118	26761	1.56
miR-369-3p	14441	22857	1.58
miR-197	20128	32193	1.60
miR-21*	12420	19874	1.60
miR-328	18750	30348	1.62
miR-471	12420	20167	1.62
miR-207	21638	38458	1.78
miR-667	17689	36156	2.04

**Mean**	**16083**	**26628**	**1.61**

Levels of miRNAs were measured with microarrays as described in the method section. Relative values of signals for each miRNAs are presented in the two columns.

**Table 5:** Hepatic miRNAs levels reduced in maternal HF fed offspring

**miRNAs**	**C/C**	**HF/C**	**↓ Fold**
miR-410	18072	11997	1.51
miR-804	19831	13138	1.51
miR-323-5p	17402	11500	1.51
let-7c	108628	71343	1.52
miR-302a*	16365	10647	1.54
miR-711	25823	16057	1.61
miR-26a	95209	58142	1.64
miR-122	324798	192912	1.68
miR-216b	16179	9463	1.71
miR-294*	17402	10168	1.71
miR-185	27121	15378	1.76
miR-192	80361	44865	1.79
miR-29a	30002	16331	1.84
miR-194	92178	49817	1.85
miR-145	28108	14841	1.89
miR-126-3p	41868	20020	2.09
miR-762	76284	32872	2.32
miR-16	109631	43004	2.55
miR-1224	69200	24241	2.85
miR-22	130448	44304	2.94
miR-30c-2*	67031	20460	3.28
miR-494	298733	82842	3.61
miR-483*	366458	74387	4.93

**Mean**	**90310**	**38640**	**2.16**

Levels of miRNAs were measured with microarrays as described in the method section. Relative values of signals for each miRNAs were presented in the two columns.

Among those miRNAs showing reduced expression, average levels of expression were 90310 arbitrary units (Table 5), whereas in those showing increased expression, the average levels of the 10 miRNAs were 16083 arbitrary units (Table 4), which was 5.6-fold lower than those miRNAs showing reduced expression.

We validated microarray data with the stem-loop RT-PCR method [49] using purchased miRNA primers (ABI). 5 miRNAs (let-7c, miR-483*, miR-22, miR-29a and miR-30c) were measured as these miRNAs showed different magnitude of reduced expression in the HF offspring (Table 6). Expression of miR-483*, let-7c and miR-29a measured with qPCR were consistent with values obtained from microarray data, with minor differences in the magnitude of changes in expression (Table 6). However, a discrepancy in levels of miR-30c and miR-22 between qPCR and microarray was observed (Table 6). Levels of miR-30c between maternal HF and chow fed offspring were similar when measured with microarrays, but significantly different when measured with qPCR. A ~2.9-fold reduction was obtained with microarray analyses whereas a ~42% increase occurred in levels of miR-22 in maternal HF offspring measured with qPCR (Table 6). We also noted that levels of miR-483* were very low when measured with qPCR, consistent with poorly expressed *Igf2 *mRNA levels. However, data from microarray suggested that miR-483* was abundantly expressed, which was not consistent with qPCR data (Table 6).

**Table 6:** Validation of microarray data with stem-loop real-time qPCR

**Genes**	**qPCR (arbitrary units)**		**Microarray**		**↓ Fold (HF/C v C/C)**	
	**C/C (n = 7)**	**HF/C (n = 7)**	**C/C**	**HF/C**	**qPCR**	**Microarray**
	
***Stem-loop real time PCR***						
**miR-30c**	1572 ± 670	1168 ± 230*	19436	18861	1.35	1.03
**miR-22**	309 ± 163	440 ± 179*	130448	44304	0.70	2.94
**miR-29a**	370 ± 117	276 ± 109*	30002	16331	1.34	1.84
**Let-7c**	330 ± 31	227 ± 19*	108628	71343	1.45	1.52
miR-483*	1.9 ± 0.2	0.3 ± 0.1***	366458	74387	6.89	4.93
***Poly dT adaptor qPCR method***						
**miR-709**	1304 ± 118	768 ± 129**	1387121	1258883	1.70	1.10
**miR-122**	948 ± 61	473 ± 40***	324798	192912	2.00	1.68
**miR-192**	321 ± 24	121 ± 10***	80361	44865	2.64	1.79
**miR-194**	124 ± 11	81 ± 7**	92178	49817	1.53	1.85
**miR-26a**	157 ± 17	58 ± 10***	95209	58142	2.72	1.64
**Let-7c**	118 ± 10	60 ± 7***	108628	71343	1.96	1.52
**let-7a**	279 ± 16	108 ± 12***	60650	43004	2.59	1.41
**let-7b**	107 ± 5	46 ± 4***	83730	63907	2.34	1.31
**let-7d**	165 ± 16	80 ± 8***	91702	72642	2.05	1.26
**miR-494**	12 ± 2	5.5 ± 0.8***	298733	82842	2.18	3.61
**miR-483**	1.3 ± 0.2	1.0 ± 0.2	17689	22100	0.78	0.80

Levels of miRNAs were measured either with purchased primers for specific miRNAs (stem-loop real time PCR) or poly dT adaptor as described in the method section. Mean ± S.E. * p < 0.05 and *** p < 0.001 (HF/C v C/C). ** p < 0.01, and ***p < 0.001 (HF/C v C/C).

We examined the miR-483* genomic DNA location because miR-483* showed the greatest reduction in expression in maternal HF fed offspring, and found that miR-483* was encoded in an intron of *Igf2*. As intronic miRNAs may share common promoters as their host genes, many intronic miRNAs show significantly correlated expression profiles with their host genes[50,51]. Thus, we would expect that the levels of intronic miRNA (e.g. miR-483*) are increased with the host gene (*Igf2*) in the HF/C mice. To our surprise, expression of miR-483* in the HF/C was reduced (shown by qPCR and microarray, Table 4a) in association with increased *Igf2 *levels. Mir-483 is also processed from the same mmu-mir-483 gene and share most of the complementary sequence of miR-483* [52]. We then examined expression levels of miR-483 from microarray, and found that expression of miR-483 was not markedly different in HF/C mice compared to the controls (Additional file 1: Table S1 and Table 6). We could not validate levels of miR-483 with the stem-loop qPCR because the primers for miR-483 were not available at the time of the study (ABI). Therefore, we measured expression of miR-483 with another qPCR method involving reverse-transcribed poly(T) adaptor during the RT step [53], and found that the low levels of miR-483 expression were consistent with microarray data, similar to poorly expressed miR-483* obtained with the stem-loop method (Table 6). We repeated measurements of let-7c using the poly (T) adaptor method and the results were consistent with those obtained from microarray or the stem-loop qPCR (Table 6). We therefore carried out further validation of miRNAs with the poly (T) adaptor method as this methodology provided flexibility in primer design.

We tried to validate miRNAs showing increased expression with the poly (T) adaptor method. However, among those showing increased expression measured with microarray, miR-667, miR-207, miR-197, miR-770-3p and miR-369-3p were very poorly expressed (data not shown). miR-328 was expressed at much higher levels, but a reduced rather than increased expression in maternal HF diet fed offspring was observed (data not shown). We then focused our study on those miRNAs showing reduced expression in maternal HF fed offspring but excluded those poorly expressed miRNAs (Table 5) plus miR-709, as microarray data suggested that miR-709 had the greatest level of expression in the liver (Additional file 1: Table S1 and Table 6). Data from qPCR confirmed the highly expressed miR-709, but also showed marked reduction in expression in maternal HF fed offspring (p < 0.01, Table 6), which was not consistent with microarray results. However, the levels of most miRNA expression measured with qPCR were consistent with data obtained from microarrays except minor differences in the magnitude of changes (Table 6).

### Bioinformatic analysis of predicted targets for miRNAs

As miR-709 was the highest expressed miRNA in the liver, it might be an important miRNA for the regulation of hepatic gene expression. We analysed the predicted targets with widely used algorithms. 1241 hits were found using the miRanda algorithm [54], whereas 353 targets were found using the TargetScan algorithm [12]. At the time of writing, miR-709 was not in the data base of PicTar [55]. We compared the outcome from miRanda and TargetSan algorithms using our own purpose-built computer program and found that 28 common targets (Additional file 2: Table S2) were predicted by both algorithms.

A feature of miRNA function is that several miRNAs tend to act together to generate greater effects than single miRNA [13]. We therefore undertook bioinformatics analysis to investigate whether it was possible to identify common targets for those validated miRNAs that showed reduced expression in the maternal HF fed offspring using our own purpose-built computer program. We found that ZSWIM3 (zinc finger, SWIM domain containing 3), a protein whose function was yet to be characterised [56], was targeted by 5 miRNAs namely miR-122, miR-192, miR-194, miR-709 and miR-483*. 14 genes were targeted by 3 different miRNAs ' [see Additional file 22: Table S3]' and 10 genes (including citrate synthase and Igf-1 receptor) were targeted by miR-122 and miR-494 ' [see Additional file 2: Table S4]'. These results suggested that functions of specific genes might be co-ordinately regulated by a small number of miRNAs.

## Discussion

Maternal HF fed offspring mice have: 1) increased hepatic expression of key genes including those regulating fetal growth (such as *Igf2*) and fat metabolism (such as *ppar-α *and *cpt-1a*); 2) altered expression of a small percentage (~5.7%) of important miRNAs. Among the miRNAs showing reduced expression, let-7c regulates developmental timing [46,47] and miR-122 regulates fat oxidation [21,57]. Thus, these data suggest co-ordinated changes in key metabolic genes and miRNAs that regulate early fetal growth and fat metabolism in offspring of dams fed HF diet.

As both offspring from HF- and chow-fed dams are weaned onto the same chow diet and maintained on chow until adulthood, changes in expression of key metabolic genes and miRNAs in adult offspring are likely to occur prior to weaning. IGF2 is a growth factor highly expressed during early development [58]. Offspring *Igf2 *gene expression can be altered by maternal dietary modifications during early development. For example, a maternal low protein diet restricted only to the preimplantation period reduces hepatic *Igf2 *mRNA in fetal rats [59] and maternal dietary calorie restriction increases *Igf2 *mRNA levels in the liver and skeletal muscle in fetal sheep [60]. Here we further show that hepatic mRNA levels of *Igf2 *are elevated in the adult mouse offspring born to dams fed a HF diet, suggesting that maternal HF feeding increases offspring hepatic *Igf2 *expression prior to weaning. This is supported by our observation that hepatic *Igf2 *levels in fetal offspring from dams fed a HF diet are increased (unpublished data). Similarly, it is likely that altered expression of hepatic *ppar-α *and *cpt-1a *and miRNAs in maternal HF fed adult offspring might have also occurred prior to weaning.

### Growth

**Figure 1:**
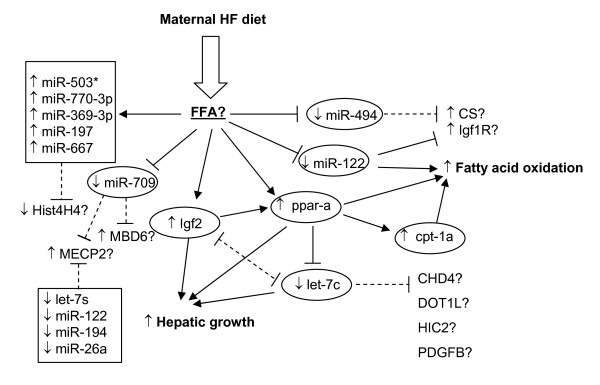
**Maternal high fat diet during gestation and lactation alters hepatic expression of key genes and miRNAs in the offspring**. A maternal HF diet during gestation and lactation increased hepatic *Igf2 *expression in the offspring, which may be required for the up-regulation of *ppar-α*/cpt-1a by HF diet as suggested by our data presented in the Table 2. Increased *ppar-α *suppresses expression of let-7c, facilitates hepatic growth. Igf2 could down regulate let-7c through increased expression of *ppar-α*. Increased expression of *ppar-α *and reduced expression of miR-122 may increase hepatic fatty acid oxidation in the offspring. Igf1 receptor (Igf1R) and citrate synthase (CS) are predicted targets shared by both miR-122 and miR-494. Inhibition of Igf1R has been confirmed very recently [86]. Similar to miR-122, maternal HF offspring have reduced miR-494 levels, which favour increased Igf1R and CS activities. Several key proteins involved in epigenetics are predicted targets for miRNAs, in particular, methyl-CpG binding protein 2 are predicted targets for 5 miRNAs (miR-709, let-7s, miR-122, miR-194 and miR-26a) showing reduced levels in maternal HF fed offspring. Histone 4 H4 are predicted targets for 5 miRNAs (miR-503*, miR-770-3p, miR-369-3p, miR-197 and miR-667) showing increased levels in maternal HF fed offspring. Arrows suggest stimulatory and blocked arrows inhibitory effects. Solid lines represent established relationships whereas broken lines represent relationships not yet confirmed experimentally. FFA: free fatty acids. CS: citrate synthase, ppar-a: peroxisome proliferator activated receptor-alpha, cpt: carnitine pamitoyltransferase, MBD: methyl-CpG binding domain protein, MECP2:Methyl-CpG-binding protein 2, CHD4:chromodomain helicase DNA binding protein 4, DOT1L: DOT1-like, histone H3 methyltransferase, HIC2: hypermethylated in cancer 2, Hist4H4, histone 4 H4.

IGF2 is an early growth factor expressed at the two-cell stage in the mouse embryo[61], and mice deficient in IGF2 have reduced birth weight [58]. In contrast, excess IGF2 in transgenic mice promotes fetal overgrowth resulting in increased birth weight [62]. Our data showing that HF/C offspring have increased *Igf2 *expression and a trend toward increased liver weight [~16% increase, compared with the control mice (p = 0.44)], suggest that liver growth prior to weaning may be increased in maternal HF fed offspring (Fig 1). This finding is consistent with an early observation in rats showing that maternal HF feeding during gestation increases offspring liver weight, in association with increased body weight and percentage body fat at weaning[38]. In our study, the body weight of maternal HF fed offspring at weaning is ~19.6% (p < 0.05) greater than control mice (data not shown), consistent with increased hepatic *Igf2 *expression. However, we observed no significant difference in body weights between maternal HF and chow fed adult offspring, when both sets of offspring were fed the same chow diet from weaning, which could be due to that circulating IGF2 is markedly decreased after birth in rodents [63,64], suggesting that IGF2 is unlikely to play a major role in post weaning growth.

PPARα promotes hepatic proliferation through inhibition of let-7c [65,66]. Let-7c plays a critical role in the regulation of growth[67]. Overexpression of let-7c decreases c-myc and miR-17, suppressing the growth of hepatocytes [66]. Consistently, we have observed that mRNA levels of *ppar-α *(and protein [33]) are elevated whereas levels of let-7c reduced in maternal HF fed offspring (Fig. 1), suggesting a co-ordinated regulation of mRNA and miRNA expression in favour of promoting hepatic growth.

It is uncertain whether let-7c is regulated by IGF2. Our data showing increased *Igf2 *expression in association with reduced let-7c expression, is consistent with the negative regulation of let-7c by PPAR-α as discussed above. In *Igf2 *KO mice, expression of let-7c levels are increased by ~31% (p = 0.004). These data suggest a negative correlation between *Igf2 *and let-7c expression (Fig. 1). Further studies are required to determine whether let-7c expression is directly regulated by IGF2. Taking together, our data suggest that maternal HF feeding has induced co-ordinated changes in expression of early growth factor (e.g. *Igf2*), transcription factor (e.g. *ppar-α*) and miRNA (e.g. let-7c) to promote hepatic growth.

### Fat metabolism

PPAR-α is a master transcription factor regulating hepatic fatty acid oxidation [68-73]. We have shown previously that maternal high fat high protein and low carbohydrate diet-fed offspring have increased protein levels of PPAR-α and CPT-1a in association with reduced hepatic lipid levels [33]. Here we further show that mRNA levels of *ppar-α *and *cpt-1a *are increased in the maternal HF fed offspring, suggesting that maternal HF feeding increases expression of *ppar-α *and *cpt-1a *mRNA and protein levels.

IGF2 may also regulate fat metabolism as low circulating IGF2 concentrations are associated with weight gain and obesity [43], whereas high circulating IGF2 levels are associated with low body weight in middle aged men [44]. Mice overexpressing *Igf2 *have increased fatty acid oxidation [45]. Our data show that maternal HF offspring have increased *Igf2 *expression with parallel increased *ppar-α*, whereas a HF induced increase in *ppar-α *expression is suppressed in the *Igf2 *KO mice. These data suggest that *Igf2 *might regulate fat metabolism through regulation of *ppar-α *expression, and that up-regulation of *ppar-α *in maternal HF diet fed offspring is mediated, at least in part through increased expression of *Igf2*.

miR-122 is abundantly expressed in the liver and regulates fat metabolism [21], as knocking down miR-122 increases hepatic fatty-acid oxidation [21,57]. Hepatic expression of miR-122 is reduced in the maternal HF adult offspring (Fig. 1), which is consistent with increased expression of PPARα and CPT-1a, two key molecules regulating hepatic fatty acid oxidation (Fig. 1). Maternal HF fed adult offspring have reduced hepatic lipid levels when weaned onto a chow diet and maintained on the chow diet until adulthood[33]. It is likely that increased capacity of fat oxidation (due to reduced miR-122 and increased PPARα and CPT-1a) prior to weaning are maintained until adulthood. This continuing increased fatty acid oxidation capacity leads to reduced hepatic lipid levels when HF offspring mice are weaned onto a chow diet. Taking together, our data suggest that maternal HF feeding increases expression of key genes regulating hepatic fatty acid oxidation and miRNA in the offspring.

The mechanisms by which early changes are maintained until adulthood require further studies. However, it is likely that epigenetic mechanisms may play an important role. *Igf2 *expression is regulated by DNA methylation[74], and increased *Igf2 *expression is associated with changes in DNA methylation [75]. Gestational choline deficiency causes global and *Igf2 *gene DNA hypermethylation through up-regulation of Dnmt1 expression in fetal offspring [75]. Thus, it is likely that increased *Igf2 *expression in maternal HF adult offspring is associated with altered DNA methylation in the offspring. Similar mechanisms may exist in *ppar-α*, because hepatic expression of *ppar-α *is regulated by DNA methylation [76]. A maternal low protein diet during pregnancy and lactation reduces DNA methylation in the promoter region of *ppar-α *[77], in association with increased *ppar-α *mRNA levels [77]. Expression of miRNAs can also be regulated by epigenetic mechanisms. Let-7a-3 belongs to the let-7 miRNA gene family and is heavily methylated by the DNA methyltransferases Dnmt1 and Dnmt3b [78]. Let-7a-3 hypomethylation facilitates epigenetic reactivation of the gene and elevates expression of let-7a-3 in human lung cancer cells [78]. Thus, altered DNA methylation could be involved in early changes maintained until adulthood.

Interestingly, several important proteins involved in epigenetics are predicted targets for those miRNAs showing altered expression in the HF offspring. For example, miR-709 is the most abundantly expressed miRNA in the liver detected with microarray (greater than miR-122). According to the TargetScan algorithm [12], miR-709 targets include methyl-CpG binding domain protein 6 and methyl CpG binding protein2 (MECP2, Fig. 1). Among predicted targets of let-7c are proteins including hypermethylated in cancer 2 (HIC2), chromodomain helicase 4, DOT1-like histone H3 methyltransferase (Fig. 1).

A feature of miRNA function is that several miRNAs tend to act together, and a relatively small set of miRNAs account for most of the differences in miRNA profiles between cell lineages and tissues [52]. For example, it has been shown that expression of 4 miRNAs (miR-29a, miR-29b, miR-29c and miR-150) is up-regulated [23], whereas expression of 11 miRNAs (miR-379, miR-127, miR299-5p, miR-434-3p, miR-335, miR130a, miR-19b, miR-451, miR-148a, miR-199a and miR-152) is down-regulated in skeletal muscle of type 2 diabetic rats [23]. Here we show that levels of only ~5.7% of miRNAs are altered in the maternal HF fed mouse offspring, whereas levels of the remaining miRNAs are unchanged. These data suggest that 1) these 23 miRNAs are likely to be expressed during early development and play active roles in the regulation of metabolism and fetal growth; and 2) if these miRNAs have common targeted transcripts, they are likely to have greater effects than a single miRNA in suppressing protein synthesis [13].

However, it remains a challenge to identify common targets shared by several miRNAs, because several hundreds or even over 1000 predicted targets may arise from one single miRNA using current algorithms. Experimentally, it is impractical to knock down each of the miRNAs. In this study, we have written a computer program that allows us to analyse quickly common targets shared by several miRNAs. For example, we have undertaken analysis of common targets among 11 miRNAs and found that the maximum number of shared targets is 5 miRNAs and no common targets are found among 6 different miRNAs. MECP2 is a common predicted target for 5 miRNAs including two abundantly expressed miRNAs (miR-709 and miR-122, Fig. 1). MeCP2 is required to maintain CpG status of genomic DNA[79,80]. Maternal nutrient restriction decreases MeCP2 levels in the brain in offspring rats[81]. Among those miRNAs showing increased expression in the HF fed offspring, histone 4 H4 is a common target for 5 different miRNAs (miR-503*, miR-770-3p, miR-369-3p, miR-197 and miR-667, Fig. 1).

Finally, despite the observation that offspring born to dams fed a HF diet during pregnancy and lactation and fed a chow diet from weaning have no significant changes in phenotype compared to the control animals, marked changes in expression of important genes such as *Igf2*, *ppar-α *and *cpt-1a *and a class of miRNAs have occurred. Such altered expression of metabolic genes and miRNAs are likely to affect the homeostatic responses of such offspring to dietary challenges in later life.

## Conclusion

A maternal HF diet prior to conception, during pregnancy and lactation induces coordinated and long-lasting changes in expression of *Igf2 *and key fat metabolic genes and miRNAs in the offspring, which may have long-term effects on their health.

## Methods

### Animal

All procedures in this study were carried out in accordance with the UK Animal Scientific Procedures Act of 1986 and approved by a local ethics committee. Female C57 BL6J black mice were maintained under controlled conditions (room temperature at 22 ± 2°C; 12 hr light/dark cycle) and randomly assigned to either a HF (22.6% fat, 23% protein and 48.6% carbohydrate, W/W) or standard chow diet (10% fat, 18% protein and 68.8% carbohydrate, W/W [82,83], RM1 - special diet services) diet. They were provided with water *ad-libitum*. Dams were fed either the HF or chow diet 4 weeks prior to conception, during pregnancy (day 1 of pregnancy indicated by presence of copulation plug) and lactation. Litter size were standardised to 6 pups. All offspring were weaned at 3 weeks of age and fed the same chow diet for 12 weeks. At 15 weeks of age, female mice (n = 7 per group from different litters) were sacrificed and liver samples were quickly removed, snap frozen in liquid nitrogen, and stored at -80°C for further analysis.

#### Igf2 KO mice

Female B6CBF1 mice were bred with male *Igf2*-knock (+/-) out mice [40]. Offspring of either WT or *Igf2 *KO mice (females only) were determined with genotyping using PCR as previously described [84]. Female animals (KO and WT) were housed individually under controlled conditions. At two months of age, female mice (both WT and KO, from different dams) were age-matched, divided into two groups, and were fed *ad-libitum *either a HF or chow diets for 6 months. Mice were sacrificed at 8 months of age, liver samples were quickly removed, snap frozen in liquid nitrogen, and stored at -80°C for further analysis.

### Preparation of total RNAs

For preparation of miRNA containing total RNAs, ~100 mg of liver tissue was homogenised in lyses buffer provided with the mirVana™ miRNA Isolation Kit (P/N: 1560, Ambion, Austin, TX) and total RNA were prepared according to the manufacturer's protocol. Purified total RNA was eluted in 100 μl of elution buffer. Concentrations of total RNAs were measured using a Nanodrop (ND-1000, NanoDrop products, Bancroft Building, Wilmington, USA). The RNA integrity was analysed using an Agilent Bioanalyser 2100 (Agilent Technologies UK Limited, Cheshire, UK) with the RNA integrity number > 8.0, and the ratio of OD_260/280 _= ~2.0.

### MiRNA microarray

This work was carried out at Febit Biomed Gmbh (Febit biomed gmbh, Heidelberg, Germany). Each sample from the control (n = 7) or HF (n = 7) offspring were pooled for microarray analysis (two-class experiment [48]). Each array contains the reverse complements of all major mature miRNAs and the mature* sequences published in the Sanger miRBase release (version 10.1, December 2007, see ) for mice. Each miRNA contains 10 replicates to increase the statistical confidence. For each array 2 μg of total RNA were labelled according to the manufacturer's instructions (miRVana labelling kit from Ambion). After labelling, samples were dried in a speed-vac and re-suspended in febit's proprietary miRNA Hybridization Buffer (18 μl per array). Samples were loaded onto a chip, and overnight (16 hours) hybridization was undertaken at 42°C, using argon pressure to move the samples within the arrays. After the hybridization, the array was washed with the 'febit miRNA standard (external incubation)' hybridization profile and a standard detection using the appropriate filter set. Data was normalised using the software "R" with the "VSN" package and are presented in Additional file 1: Table S1.

### Measurement of mRNA expression using real time PCR

During cDNA synthesis, ~200-500 ng of total RNA was used in a 20 μl cDNA synthesis reaction. Total RNA was denatured at 70°C for 5 min and chilled in ice. Then the reaction was added with random hexamers (2.5 ng/μl). The reactions were undertaken at 42°C for 60 min and the reaction was stopped by denaturing at 95°C for 5 min.

For PCR reactions, allsamples from 4 groups of animals were measured in one single 96-well plate, with each reaction undertaken in triplicates. Equal volume of cDNA (0.5 μl/reaction) was added to Sensimix Lowref SYBR green qPCR reagent (Quantace Ltd, London, UK) with gene specific primers (1.0 μM) ' [see Additional file 2: Table S5]'. PCR reactions for all samples including no temperate controls were run on a 7500 Fast Realtime PCR System (Applied Biosystems, Warrington UK, which was also used for all miRNA analysis described below). The reaction conditions were 95°C for 15 min (hotstart) at 95°C for 15 sec, 60°C for 30 sec and 72°C for 30 sec. Results were analyzed using 7500 System SDS software (v1.4). Expression levels were calculated by normalisation to a standard curve using the total amount of RNA as a denominator and expressed as arbitrary units.

### Measurement of miRNAs using real time RT-PCR

Two qPCR methods were used in the validation of microarray: the stem-loop RT-PCR method [49], using miRNA specific primers purchased from Applied Biosystems and polyadenylated and reverse-transcribed with a poly(T) adapter into cDNAs for real-time PCR using sequence complementary to the poly(T) adapters during RT reactions [53].

The stem-loop RT PCR (Taqman based technology) was performed according to the manufacturer's protocol (Applied Biosystems, Foster City, CA, USA). ~100 ng total RNA was added to each reverse transcription reaction (RT) for each miRNA. Three replicates were done for each miRNA from RT to PCR and the results were averaged.

For poly(T) adaptor RT-PCR, ~100 ng total RNA was added to a reaction containing 2.5 units E. Coli Poly A polymerase (New England Biolabs Ltd. Herts. UK), 0.75 mM rATP and 1 × Pol A polymerase buffer containing 250 mM NaCl, 50 mM Tris-HCl, 10 mM MgCl_2_. The reaction (10 μl) was incubated at 37°C for 30 min for extension of the poly A tail. The reaction was heated to 60°C for 5 min, cooling to 4°C and added with an Oligo dT adaptor (0.5 μl of 5 μM) with RT buffer, dNTP mix and MMLV and H_2_O to a total 20 μl reaction volume (Applied Biosystems, according to the manufacturers' conditions) and incubated at 42°C for 60 min for cDNA synthesis. The cDNA synthesis reaction was stopped by heating at 95°C for 5 min.

Real time PCR was undertaken with Sensimix Lowref SYBR green qPCR reagent (Quantace Ltd, London, UK) in triplicates. 0.5 μl of cDNA was added to PCR reaction containing 1 × PCR reagent mix and universal primer (0.25 μM), miRNA specific primer (0.5 μM, designed based on miRNA sequences released (Release 12.0 Sept 2008) by the Sanger Institute [85]. The reaction conditions were 95°C for 15 min (hotstart), and 95°C for 15 sec, 60-62°C for 60 sec (optimised according to each specific miRNA primers) and a total of 40 cycles.

### Computer analysis of target predictions for miRNAs

Prediction of targets for a single miRNA was undertaken using three algorithsms: TargetScan [12], miRanda [54] and PicTar [55]. To identify groups of miRNAs having common predicted targets, we have written a computer program which can be used in conjunction with any of the three algorithms. As the list of targets generated by TargetScan or miRanda are more comprehensive than those from PicTar, we based our analysis of common targets on TargetScan and miRanda.

### Statistical analysis

Data from real time PCR were presented as mean ± SE. Skewed data were transformed before statistical analysis. A student *t*-test was used to compare results between two groups, and a p value < 0.05 was considered to be significant.

## Authors' contributions

JZ and CB conceived and designed the study. JZ and FZ undertook experiments. JZ conceived the idea and XD wrote the programme for analysis of common targets prediction. JZ analysed the data and wrote the manuscript. KB and FC conducted the animal studies and prepared the liver samples. KB, FC, AC, CB, HL, MH and MV contributed to the discussion of data. All authors read and approved the final manuscript.

## Supplementary Material

Additional file 1 **Table S1.** Hepatic miRNA expression profile in adult mice. The data set provided analysed data obtained from microarray on either HF/C or C/C using pooled total RNA samples. Click here for file

Additional file 2 **Table S2, S3, S4 and S5**. S2: The table listed 28 common predicted targets shared by both TargetScan and miRbase algorithm. S3: The table listed 14 common predicted targets shared by 3 different miRNAs. S4: The table listed 11 common targets predicted by both miRNA-122a and miR-494. S5: The table listed DNA sequences for measurement of 3 mRNA transcripts. Click here for file
