# Supplementary material for: Maternal high fat diet during pregnancy and lactation alters hepatic expression of insulin like growth factor-2 and key microRNAs in the adult offspring
Source: BMC Genomics. 2009 Oct 16;10:478. doi: 10.1186/1471-2164-10-478 (PMC2770530; doi:10.1186/1471-2164-10-478)
Supplement: Additional file 2 — Table S2, S3, S4 and S5. S2: The table listed 28 common predicted targets shared by both TargetScan and miRbase algorithm. S3: The table listed 14 common predicted targets shared by 3 different miRNAs. S4: The table listed 11 common targets predicted by both miRNA-122a and miR-494. S5: The table listed DNA sequences for measurement of 3 mRNA transcripts. [file 1471-2164-10-478-S2.DOC]

Additional tables

**Additional table 2. Common predicted target for miR-709 by TargetScan and miRbase algorithm**.

| **Gene symbol** | **Gene description** |
| --- | --- |
| AP2S1 | adaptor-related protein complex 2, sigma 1 subunit |
| ARAF | Araf v-raf murine sarcoma 3611 viral oncogene homolog |
| BCL9L | Bcl9l B-cell CLL/lymphoma 9-like |
| BRUNOL4 | bruno-like 4, RNA binding protein |
| CAMK2B | Camk2b calcium/calmodulin-dependent protein kinase II-β |
| CECR5 | cat eye syndrome chromosome region, candidate 5 homolog |
| CNN3 | calponin 3, acidic |
| DNAJC19 | DnaJ (Hsp40) homolog, subfamily C, member 19 |
| ERBB4 | v-erb-a erythroblastic leukemia viral oncogene homolog 4 (avian) |
| GNPTAB | N-acetylglucosamine-1-phosphate transferase, α and β subunits |
| GPR162 | G protein-coupled receptor 162 |
| HIPK4 | homeodomain interacting protein kinase 4 |
| HNRPUL1 | heterogeneous nuclear ribonucleoprotein U-like 1 |
| IMPDH1 | inosine 5'-phosphate dehydrogenase 1 |
| IQGAP2 | IQ motif containing GTPase activating protein 2 |
| JOSD2 | Josephin domain containing 2 |
| KRT80 | keratin 80 |
| LRP6 | low density lipoprotein receptor-related protein 6 |
| NAB2 | Ngfi-A binding protein 2 |
| NRF1 | nuclear respiratory factor 1 |
| NRIP2 | nuclear receptor interacting protein 2 |
| PACS1 | phosphofurin acidic cluster sorting protein 1 |
| SAMD4B | sterile alpha motif domain containing 4B |
| SH3RF2 | SH3 domain containing ring finger 2 |
| SLC36A1 | solute carrier family 36 (proton/amino acid symporter), member 1 |
| SPATA18 | spermatogenesis associated 18 |
| TIAL1 | cytotoxic granule-associated RNA binding protein-like 1 |
| USF2 | upstream transcription factor 2 |

1241 hits were found with miRbase algorithm, whereas 353 conserved targets are found using the TargetScan algorithm for mmu-miR-709. 28 common targets are found between the two algorithms using our purpose-built programme.

**Additional table 3. Common predicted targets shared by 3 miRNAs.**

| **Gene symbol** | **Gene description** | **Targeted by miRNAs** |
| --- | --- | --- |
| 2810432D09RIK | RIKEN cDNA 2810432D09 gene | miR-194,192 and 26a |
| AHNAK | AHNAK nucleoprotein (desmoyokin) | miR-709,192 and 26a |
| B3GALTL | beta 1,3-galactosyltransferase-like | miR-709, 194 and 192 |
| DAD1 | defender against cell death 1 | miR-709, 194 and 26a |
| DARS | aspartyl-tRNA synthetase | miR-194, 192 and 26a |
| DMRT1 | doublesex and mab-3 related transcription factor 1 | miR-194, 192 and 26a |
| DNAJC12 | DnaJ (Hsp40) homolog, subfamily C, member 12 | miR-194, 192 and 26a |
| LUC7L | Luc7 homolog (S. cerevisiae)-like | miR-194, 192 and 709 |
| NSL1 | NSL1, MIND kinetochore complex component, homolog (S. cerevisiae) | miR-709, 194 and 192 |
| OTUD7A | OTU domain containing 7A | miR-709, 192 and 26a |
| PPM1M | protein phosphatase 1M | miR-709, 194 and 26a |
| PPWD1 | peptidylprolyl isomerase domain and WD repeat containing 1 | miR-194, 192 and 26a |
| SNX1 | sorting nexin 1 | miR-709, 194 and 192 |
| ZSWIM3 | zinc finger, SWIM domain containing 3 | miR-709, 194 and 192 |

Among 11 miRNA showing reduced expression in the maternal HF fed offspring, lists of targets predicted with the TargetScan algorithm for each miRNA were analysed using our own programme. 14 common targets were found to be shared by three different miRNAs.

**Additional table 4. Common transcripts targeted by both miR-122a and miR-494.**

| **Gene symbol** | **Gene description** |
| --- | --- |
| ADAM10 | a disintegrin and metallopeptidase domain 10 |
| BIRC4 | X-linked inhibitor of apoptosis |
| CS | Citrate synthase |
| FUT8 | fucosyltransferase 8 |
| G3BP2 | GTPase activating protein (SH3 domain) binding protein 2 |
| IGF1R | insulin-like growth factor I receptor |
| MIPOL1 | mirror-image polydactyly gene 1 homolog |
| NEGR1 | neuronal growth regulator 1 |
| NFAT5 | nuclear factor of activated T-cells 5 |
| PURB | purine rich element binding protein B |
| TMEM32 | transmembrane protein 32 |

**Additional table 5. Primer sequences for mRNA measurement with real time qPCR**

| **Genes**  **(A/C No)** |  | **Primer sequences** |
| --- | --- | --- |
| IGF2 | Fward | ggaagtcgatgttggtgctt |
| (NM_010514) | Reverse | tgaaggcctgctgaagtagaa |
| PPARα | Fward | ctctgggcaagagaatccac |
| (NM_011144) | Reverse | tgatgtcacagaacggcttc |
| CPT-1a | Fward | tgtttcgacaggtggtttga |
| (NM_013495) | Reverse | gaagagccgagtcatggaag |
